# Association of complement components with the risk and severity of NAFLD: A systematic review and meta-analysis

**DOI:** 10.3389/fimmu.2022.1054159

**Published:** 2022-12-07

**Authors:** Jianbo Zhao, Yafei Wu, Peng Lu, Xiaoqin Wu, Junming Han, Yingzhou Shi, Yue Liu, Yiping Cheng, Ling Gao, Jiajun Zhao, Zhen Wang, Xiude Fan

**Affiliations:** ^1^ Clinical Medical College, Ningxia Medical University, Yinchuan, Ningxia, China; ^2^ Department of Endocrinology, Shandong Provincial Hospital Affiliated to Shandong First Medical University, Jinan, Shandong, China; ^3^ Shandong Clinical Research Center of Diabetes and Metabolic Diseases, Jinan, Shandong, China; ^4^ Shandong Key Laboratory of Endocrinology and Lipid Metabolism, Jinan, Shandong, China; ^5^ Shandong Prevention and Control Engineering Laboratory of Endocrine and Metabolic Diseases, Jinan, Shandong, China; ^6^ Shandong Engineering Research Center of Stem Cell and Gene Therapy for Endocrine and Metabolic Diseases, Jinan, Shandong, China; ^7^ Department of Inflammation and Immunity, Cleveland Clinic, OH, Cleveland, United States

**Keywords:** nonalcoholic fatty liver disease, complement components, disease progression, disease severity, biomarker, meta-analysis

## Abstract

**Background:**

It is generally believed that complement system is strongly associated with the risk of nonalcoholic fatty liver disease (NAFLD). However, complement system contains a variety of complement components, and the relationship between complement components and the risk and severity of NAFLD is inconsistent. The aim of this meta-analysis was to evaluate the association of complement components with the risk and severity of NAFLD.

**Methods:**

We searched PubMed, Embase, Cochrane Library, Google Scholar, Scopus, and ZhiWang Chinese databases from inception to May 2022 for observational studies reporting the risk of NAFLD with complement components. Random-effects meta-analysis was used to obtain pooled estimates of the effect due to heterogeneity.

**Results:**

We identified 18 studies with a total of 18560 included subjects. According to recent studies, levels of complement component 3 (C3) (mean difference (MD): 0.43, 95% confidence interval (CI) 0.26-0.60), complement component 4 (C4) (MD: 0.04, 95% CI 0.02-0.07), complement component 5(C5) (MD: 34.03, 95% CI 30.80-37.27), complement factor B (CFB) (MD: 0.22, 95% CI 0.13-0.31) and acylation stimulating protein (ASP) (standard mean difference (SMD): 5.17, 95% CI 2.57-7.77) in patients with NAFLD were significantly higher than those in the control group. However, no statistical significance was obtained in complement factor D (CFD) levels between NAFLD and non-NAFLD (MD=156.51, 95% CI -59.38-372.40). Moreover, the levels of C3, C5, CFB, and ASP in patients with moderate and severe NAFLD were significantly higher than those in patients with mild NAFLD. Except for C4 and CFD, the included studies did not explore the changes in the severity of NAFLD according to the concentration of C4 and CFD.

**Conclusions:**

This meta-analysis demonstrates that an increase in complement components including C3, C5, CFB, and ASP is associated with an increased risk and severity of NAFLD, indicating that they may be good biomarkers and targets for the diagnosis and treatment of NAFLD.

**Systematic review registration:**

PROSPERO [https://www.crd.york.ac.uk/PROSPERO/], identifier CRD42022348650.

## Introduction

As one of the most prevalent chronic liver diseases in the world, nonalcoholic fatty liver disease (NAFLD) has become a major public health problem in recent years, and its overall morbidity in America is approximately 10-30% (1). Without timely treatment, the liver may gradually progress to cirrhosis, and even hepatocellular carcinoma and liver failure, thus seriously affecting the patient’s quality of life and longevity (2). Liver steatosis and fibrosis in NAFLD involve many risk factors, such as obesity, diabetes, and age > 50 years. However, the specific pathological mechanism for the involvement of these factors during disease progression remains unclear (3–5).

Previous evidence has confirmed that multiple extrahepatic organs and a variety of regulatory pathways are involved in the development of NAFLD (3). Moreover, multiple cytokines or chemokines secreted by immune cells and parenchymal cells are involved in the occurrence and development of NAFLD, such as the complement system. It is composed of complement main components, humoral regulatory factors, and complement receptors (6, 7). The complement system is a highly regulatory protein reaction system, including more than 60 components, which are widely distributed in serum, tissue fluid and cell membrane surface. Until now, three main complement activation pathways (classical pathway, alternative pathway and lectin pathway) have been found (8). There are other pathways, such as the activation of the intracellular complement system by direct cleavage of C3 and C5 by intracellular proteases (9). Complement intrinsic components (complement component 1 (C1)-complement component 9 (C9), etc), complement receptors (CR1-CR5, etc) and complement regulatory proteins (complement factor H (CFH) and complement factor I (CFI), etc) are widely involved in the activation and regulation of complement system (8, 10). As one of the components in the immune system, complement plays a critical role in defense and a surveillance function through rapid and extensive responses to microorganisms, apoptotic cells, and other threats (11). Complement is widely involved in inflammation, obesity, diabetes, insulin resistance (IR), neurodegenerative diseases, and cancer (12). Activation and inhibition of the complement system occur simultaneously and restrict each other, while regulators and inhibitors work together to ensure cell and tissue integrity. The complement system is widely activated in NAFLD and is associated with the development and severity of NAFLD (13). However, the complement system contains a variety of complement components, and the mechanism and changes of various complement components in NAFLD or nonalcoholic steatohepatitis (NASH) have not been well investigated (14).

Studies have demonstrated that an increase in C3 and C5, two of the central components of the complement system, was significantly correlated with the risk of NAFLD (15). As an independent influencing factor for NAFLD, C3 can accurately predict the presence of NAFLD (16, 17). The level of C3 in the plasma of NAFLD patients was increased, which was mainly related to insulin resistance (18, 19). Complement component 3 a (C3a) was associated with hepatic steatosis and hepatocellular injury in humans (20, 21). In addition, C5 promoted the deposition of liver triglycerides (TGs) to some extent and increased the occurrence of liver steatosis and inflammation (6, 7). Compared with the lean control group, the serum complement component 5 a (C5a) concentration was higher in obese children with NAFLD (22). Inhibition of C3 can improve the function of mouse macrophages and reduce cholestatic liver injury (20, 23). The levels of C3 and C3aR (C3a receptor) in adipose tissue of mice fed a high-fat diet increased, but the expression of C3aR in liver did not change significantly (24). C3aR-deficient mice were protected from HFD-induced hepatic steatosis (24). Some studies have found that the use of C3aR and C5aR (C5a receptor) antagonists can attenuate high-fat-induced obesity, insulin resistance and adipose tissue inflammation (25). However, a clinical study found that serum concentrations of C3 and C4 decreased along with increased Child−Pugh scores in cirrhosis (26). Moreover, the levels of several complement components involved in complement activation were increased in NASH patients during cirrhosis (27). Similarly, the level of ASP in the plasma of NAFLD patients was also increased, which was mainly related to insulin resistance (18, 19). Complement component 7 (C7) was positively correlated with liver fibrosis in NASH, while complement component 8 (C8) γ chain was negatively correlated with liver fibrosis in NASH (28). Short-term HFD (high-fat diet) increased the turnover rates of CFB (29). However, mRNA expression of CFB in the liver did not differ between healthy subjects and NASH subjects (30). Some studies confirmed that circulating CFD levels were positively correlated with the presence of NAFLD, other studies found that the level of circulating CFD in obese adults was negatively correlated with the risk of NAFLD (31–34). It remains controversial whether the levels of complement components (C3, C4, CFB, CFD, etc.) are elevated or decreased in NASH, and there is no authoritative evidence to support their correlation. Given the inconsistency of the current results, more research is needed to explore the association of complement components with NAFLD. Therefore, we conducted this meta-analysis to further understand whether complement components are associated with the risk and severity of NAFLD.

## Patients and methods

### Search

After a careful review of the Preferred Reporting Items for Systematic Review and Meta-Analyses (PRISMA) statement (35), we mainly searched the following electronic databases: PubMed, Embase, the Cochrane Library, Scopus, Google Scholar, and Zhiwang Chinese database up to 28 May 2022. We identified more than 60 complement components, including complement main components, modulators, receptors, cell membrane proteins and multiple activation products, etc (8, 14, 36–38). We conducted a detailed search of each complement component in each database. The following search terms were used for the title or abstract: (“fatty liver” or “NASH” or “NAFLD” or “nonalcoholic fatty liver” or “nonalcoholic steatohepatitis” or “non-alcoholic fatty liver disease” or “fatty liver, nonalcoholic”) and (“complement system proteins” or “proteins, complement system” or “complement protein” or “protein, complement” or “complement proteins” or “proteins, complement” or “complement” or “hemolytic complement” or “complement, hemolytic” or “complement factors”). The search was restricted to studies in human beings. There were no language or date restrictions. Reference lists of the retrieved articles were also examined and searched manually for additional eligible publications.

### Inclusion and exclusion criteria

We chose and included studies according to the following inclusion criteria (1): original studies that evaluated the correlation between NAFLD and complement components (2); observational studies, including but not limited to cross-sectional studies (3); participants ≥18 years old (4); the diagnosis of NAFLD was based on ultrasound, computed tomography or biopsy; and (5) at least one complement component was reported. The exclusion criteria were as follows (1): duplicated reports (2); editorials, reviews, abstracts, news, and case reports (3); animal or basic studies; and (4) no appropriate results or adequate data.

### Study selection and data extraction

Two researchers independently extracted the following information according to eligible studies (1): study: the first author, publication year, location, sample size, time period and study design (2); participants: number, age and sex of the NAFLD and non-NAFLD groups (3); diagnostic methods of NAFLD; and (4) outcome indicator. When differences arose, we discussed the data eligibility with the third author until a consensus was reached.

### Quality assessment

To ensure the reliability of the research quality, the Agency for Healthcare Research Quality (AHRQ) was used to estimate the inclusion of observational studies from 11 items. Article quality was assessed as follows: Studies of 0-3 points were classified as low quality; studies of 4-7 points were classified as medium quality; and if the study scored 8 or more points, it was considered high quality. Only studies above medium quality met our inclusion criteria.

### Statistical analysis

We evaluated the association between the complement components involved and the risk of NAFLD based on the retrieved literature and evaluated the changes in the levels of complement factors in mild, moderate, and severe NAFLD to explore the relationship between complement components and the severity of NAFLD. The results of the systematic analysis were represented by the mean difference (MD) and the corresponding 95% confidence intervals (CIs). Heterogeneity among studies was quantified using Cochran’s Q test and the I^2^ statistic. If I^2^>50%, then it was considered to have at least moderate heterogeneity, and a random effects model was used for the analysis. With the aim of identifying the sources of heterogeneity that cannot be ignored, we conducted subgroup analysis according to the region where the study was performed (Eastern and Western countries) and the type of research (cross-sectional study and case−control study). Meta-regression analyses were further carried out if there was significant heterogeneity. Sensitivity analysis was assessed by removing each study one at a time and determining its effect on the ultimate effect estimate. P < 0.05 indicated the existence of statistical significance. To determine whether there was a potential publication bias, funnel plots and Egger’s test were carried out to check for publication bias. The related statistical analyses were performed using Stata software (version 12.0) and Review Manager (RevMan, version 5.4).

## Results

### Search results and baseline characteristics of the studies

**Figure 1:**
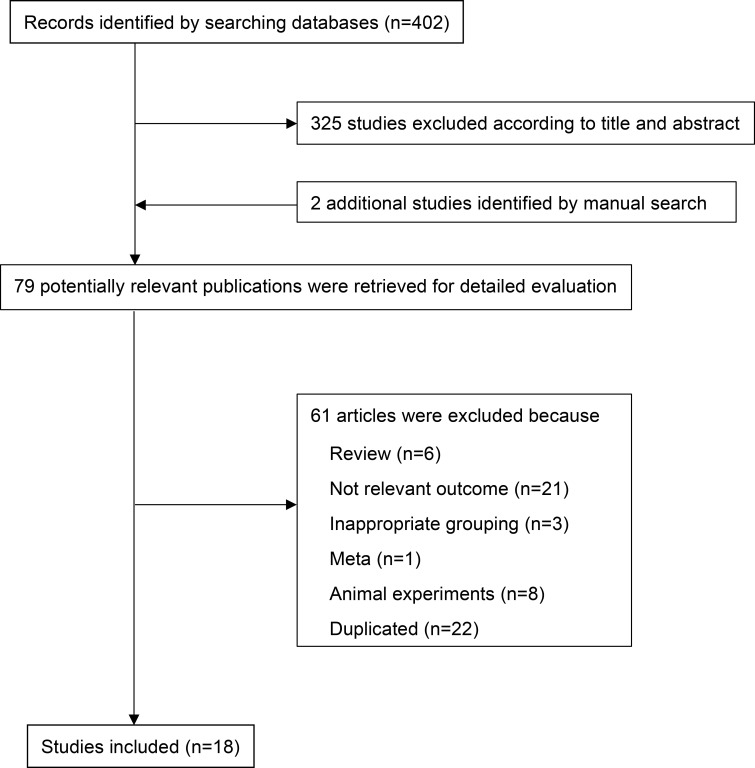
Flowchart for the search and selection of research. randomized controlled trial.

The PRISMA flow chart of the literature retrieval process is shown in **Figure 1**.** **A total of 402 potentially relevant publications were retrieved. After preliminary screening, 325 papers were deleted according to the titles and abstracts. Two additional studies were identified by manual search. By consulting the full text, 61 articles were further excluded for the following reasons (1): non-relevant outcome (2); reviews, animal experiments or not related; and (3) repeated publications. Finally, 18 eligible observational studies involving 18560 individuals (including 6647 NAFLD patients) were included in the meta-analysis (2, 12, 16, 17, 19, 21, 31–34, 39–46) (**Table 1**). We searched clinical studies reporting the relationship between complement components and NAFLD in six databases. These clinical studies mainly involved C3, ASP, C4, C5, CFB, CFD, a total of six complement components. Among them, 5 studies were case−control studies, 12 were cross-sectional studies and 1 study was cohort study. The sample size ranged from 76 to 7540 participants (communities or hospitals). Among them, 13 studies compared the level of C3 between NAFLD patients and healthy controls, and 4 studies compared the level of C3 between different degrees of NAFLD. Three studies compared the level of ASP between NAFLD patients and healthy controls, and 3 studies compared the level of ASP between different degrees of NAFLD. Five studies compared the level of C4 between NAFLD patients and healthy controls. Three studies compared the level of C5 between NAFLD patients and healthy controls, and 3 studies compared the level of C5 between different degrees of NAFLD. Three studies compared the level of CFB between NAFLD patients and healthy controls, and 2 studies compared the level of CFB between different degrees of NAFLD. Four studies compared the level of CFD between NAFLD patients and healthy controls. The articles we have retrieved mainly provide appropriate results for the above complement components, and do not involve receptor proteins and regulatory proteins. These studies were conducted in Eastern (China) and Western (Italy and Turkey) countries. In addition, most studies used ultrasound or guidelines to diagnose NAFLD, while only a few studies were based on liver biopsy (47–51). The quality scores of all the included studies were between 5 and 9 points, which were considered to be above medium quality (**Supplementary Table 1**).

**Table 1:** Basic characteristics of the included studies.

Author	Year	Location	Study design	Sample size	Age		Outcome index	Study period	Diagnosis of NAFLD
					NAFLD	Control			
Binbin Pan (17)	2020	China	Cross-sectional	648	64.92 ± 14.74	65.15 ± 17.48	C3, C4	2009.8-2012.10	Ultrasound
Chengfu Xu (2)	2016	China	Cross-sectional	7540	51 ± 14.2	48.4 ± 15.6	C3	2014	Ultrasound
Chenghua Liu (40)	2017	China	Cross-sectional	390	49.5 ± 6.4	48.3 ± 6.9	C3, C5, ASP, BF	2015.10-2017.10	Guideline
Francesco (16)	2017	Italy	Cross-sectional	164	58.7 ± 9.8	62.8 ± 10.1	C3, C4	2016.1-2016.12	Ultrasound and HSI
Guoyu Li (39)	2016	China	Cross-sectional	190	48.65 ± 6.12	47.52 ± 5.72	C3, C5, ASP, BF	2011.1-2013.1	Guideline
Jinhua Zhang (32)	2021	China	Cross-sectional	1163	53.7 ± 7.3	52.6 ± 7.2	CFD	2011-2013	Ultrasound and Liver Fibrosis
Limin Feng (12)	2021	China	Cross-sectional	3673	50.5 ± 8.9	48.7 ± 9.9	C3	2014.7-2017.11	Ultrasound
Longman Li (21)	2021	China	Cross-sectional	1600	38 (32–45)	35(28-44)	C3, C4	2009.9-2009.12	Ultrasound
Wei Wei (45)	2006	China	Cross-sectional	101	50.68 ± 11.86	43.91 ± 12.76	C3	2003.9-2005.9	Guideline
Xiaoyin Jiang (46)	2019	China	Cross-sectional	358	48.9 ± 8	45.7 ± 9.9	C3	2017.4-2018.3	Guideline
Yan Wang (43)	2011	China	Cross-sectional	80	/	/	C3	2010.1-2010.5	Guideline
Yanbin Wang (42)	2015	China	Case-control	1000	45.6 ± 7.1	/	C3, C4, C5, BF	2008.5-2013.9	Ultrasound
Yingying Gu (31)	2022	China	Cohort	908	59.96(56.60-63.48)	59.50(56.58-64.58)	CFD	2014.4-2017.5	Guideline
Yongan Liu (41)	2013	China	Case-control	180	/	/	C3, C5, ASP	/	Guideline
Yongqin Wang (44)	2016	China	Cross-sectional	104	37.3 ± 10.6	33.4 ± 12.7	C3, C4	2012.5-2014.12	Guideline
Yun Qiu (34)	2019	China	Case-control	211	33.09 ± 5.57	33.25 ± 6.66	CFD	2016.4-2016.9	Guideline
Yusuf Yilmaz (33)	2011	Turkey	Case-control	174	48 ± 8	48 ± 7	CFD	2010	Liver Biopsy
Zeki Yesilova (19)	2005	Turkey	Case-control	76	33.04 ± 9.95	33.63 ± 12.31	C3, ASP	/	Liver Biopsy

ASP, acylation stimulating protein(nmol/L); C3, complement component 3(g/L); C4, complement component 4(g/L); C5, complement component 5(mg/L); CFB, complement factor B(g/L); CFD, complement factor D(ng/mL); NAFLD, nonalcoholic fatty liver disease.

### Association of serum complement components with the risk of NAFLD

**Figure 2:**
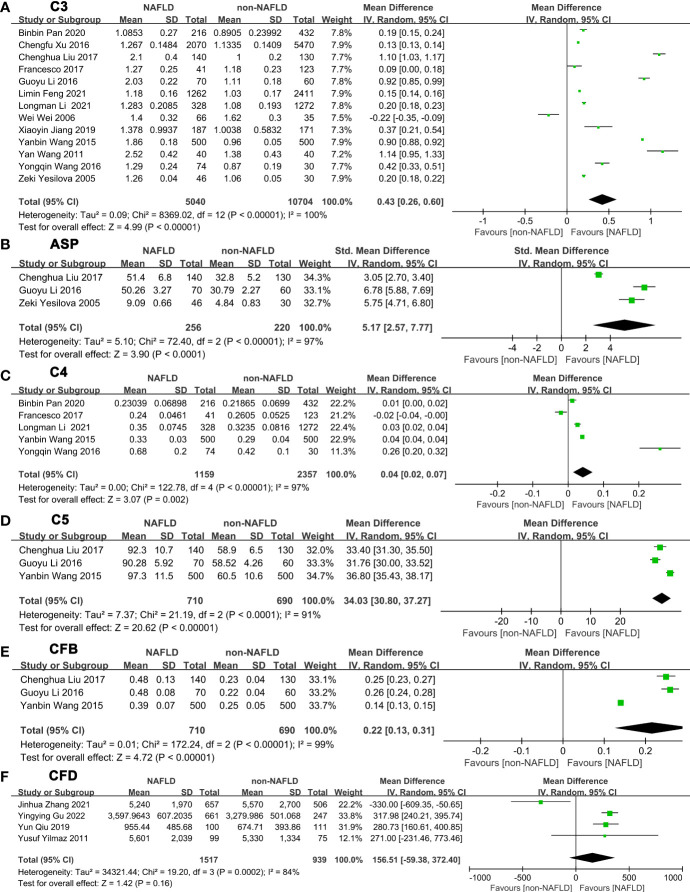
Forest plots of the deviation of complement components between NAFLD and non-NAFLD. The forest plots show the MD value and 95% CI of the NAFLD group compared with the non-NAFLD group. Due to the large heterogeneity, a random effect model was used to analyze the included studies. **(A)** A random effect model was used to analyze the deviation of C3 between NAFLD and non-NAFLD. **(B)** A random effect model was used to analyze the deviation of ASP between NAFLD and non-NAFLD. **(C)** A random effect model was used to analyze the deviation of C4 between NAFLD and non-NAFLD. **(D)** A random effect model was used to analyze the deviation of C5 between NAFLD and non-NAFLD. **(E)** A random effect model was used to analyze the deviation of CFB between NAFLD and non-NAFLD. **(F)** A random effect model was used to analyze the deviation of CFD between NAFLD and non-NAFLD. Abbreviations: ASP, acylation stimulating protein; C3, complement component 3; C4, complement component 4; C5, complement component 5; CFB, complement factor B; CFD, complement factor D; CI, confidence interval; IV, Inverse Variance; NAFLD, nonalcoholic fatty liver disease; SD, standard deviation.

After summarizing all studies, we compared expression of these complement components between NAFLD and non-NAFLD (**Figure 2** **;** **Supplementary Table 2**). Among the 14 studies included in the meta-analysis, 13 studies were able to provide appropriate C3 data to quantitatively assess the relationship between C3 and NAFLD (**Figure 2A**). The meta-analysis results showed that serum C3 levels in the NAFLD population were significantly higher than those in the non-NAFLD population (MD: 0.43, 95% CI [0.26~0.60]) (I^2 =^ 100%, P for heterogeneity <0.00001). Three studies reported appropriate data to quantitatively assess the relationship between ASP and NAFLD (**Figure 2B**). The meta-analysis results showed that serum ASP levels in NAFLD patients were higher than those in the control group (MD: 5.17, 95% CI [2.57~7.77]) (I^2 =^ 97%, P for heterogeneity<0.00001). Sensitivity analyses showed that no single study significantly affected the results.

Five studies were able to provide appropriate C4 data to quantitatively assess the relationship between C4 and NAFLD (**Figure 2C**). The meta-analysis results showed that serum C4 levels in serum of patients with NAFLD were higher than those in the control group (MD: 0.04, 95% CI [0.02~0.07]) (I^2^ = 97%, P for heterogeneity < 0.00001). In addition, 3 studies can provide appropriate C5 data to quantitatively assess the relationship between C5 and NAFLD (**Figure 2D**). C5 levels in serum of patients with NAFLD were higher than those in the control group (MD: 34.03, 95% CI [30.80~37.27]) (I^2 =^ 91%, P for heterogeneity<0.0001). Three studies were able to provide appropriate CFB data to quantitatively assess the relationship between CFB and NAFLD (**Figure 2E**). Serum CFB levels in patients with NAFLD were higher than those in the control group (MD: 0.22, 95% CI [0.13~0.31]) (I^2^ = 99%, P for heterogeneity<0.00001). Four studies were able to provide appropriate CFD data to quantitatively assess the relationship between CFD and NAFLD (**Figure 2F**). However, the meta-analysis demonstrated that no statistical significance was obtained between NAFLD and non-NAFLD in CFD level (MD=156.51, 95% CI [-59.38~372.40]) (I^2^ = 84%, P for heterogeneity=0.0002). Sensitivity analyses showed that no single study significantly changed the results.

### Association between serum complement components and the severity of NAFLD

**Figure 3:**
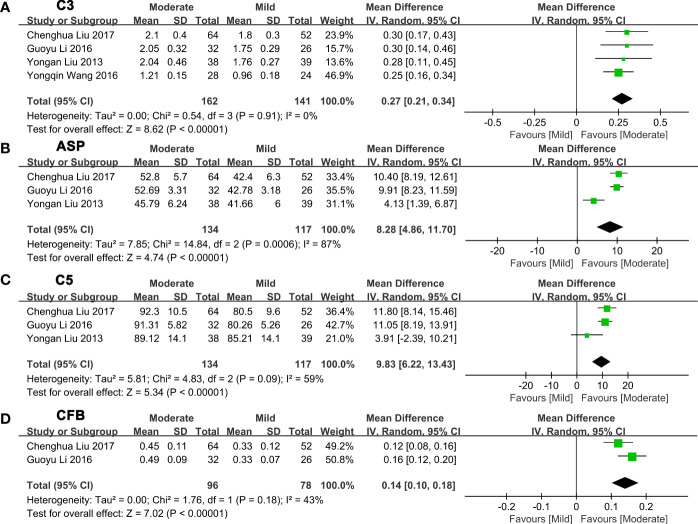
Forest plots of the deviation of complement components between mild and moderate NAFLD. **(A)** A random effect model was used to analyze the deviation of C3 between mild NAFLD and moderate NAFLD. **(B)** A random effect model was used to analyze the deviation of ASP between mild NAFLD and moderate NAFLD. **(C)** A random effect model was used to analyze the deviation of C5 between mild NAFLD and moderate NAFLD. **(D)** A random effect model was used to analyze the deviation of CFB between mild NAFLD and moderate NAFLD. ASP, acylation stimulating protein; C3, complement component 3; C5, complement component 5; CFB, complement factor B; CI, confidence interval; IV, Inverse Variance; NAFLD, nonalcoholic fatty liver disease; SD, standard deviation.

**Figure 4:**
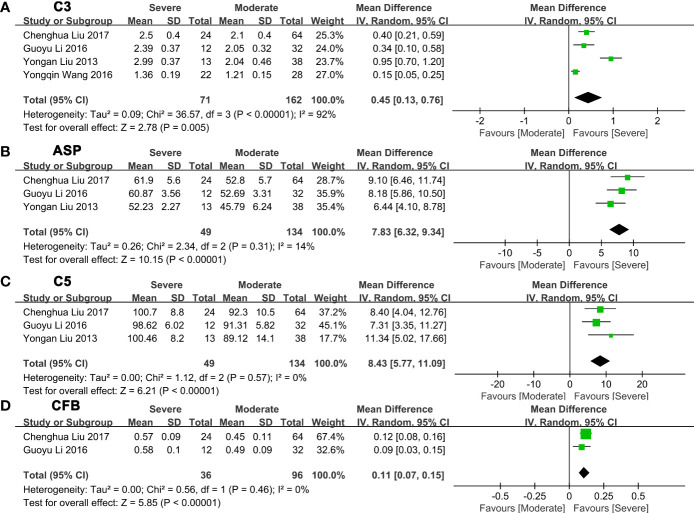
Forest plots of the deviation of complement components between moderate and severe NAFLD. **(A)** A random effect model was used to analyze the deviation of C3 between moderate NAFLD and severe NAFLD. **(B)** A random effect model was used to analyze the deviation of ASP between moderate NAFLD and severe NAFLD. **(C)** A random effect model was used to analyze the deviation of C5 between moderate NAFLD and severe NAFLD. **(D)** A random effect model was used to analyze the deviation of CFB between moderate NAFLD and severe NAFLD. Abbreviations: ASP, acylation stimulating protein; C3, complement component 3; C5, complement component 5; CFB, complement factor B; CI, confidence interval; IV, Inverse Variance; NAFLD, nonalcoholic fatty liver disease; SD, standard deviation.

Next, we made a pairwise comparison between mild, moderate, and severe fatty liver. We found that the levels of C3, ASP, C5, and CFB increased with the severity of NAFLD (**Figures 3** **-** **4** **;** **Supplementary Tables 3-4**). Serum C3 levels in moderate NAFLD were higher than those in mild NAFLD (MD: 0.27, 95% CI [0.21~0.34], P<0.00001) (**Figure 3A**); serum ASP levels in moderate NAFLD were higher than those in mild NAFLD (MD: 8.28, 95% CI [4.86~11.70], P<0.00001) (**Figure 3B**); serum C5 levels in moderate NAFLD were higher than those in mild NAFLD (MD: 9.83, 95% CI [6.22~13.43], P<0.00001) (**Figure 3C**); and serum CFB levels in moderate NAFLD were higher than those in mild NAFLD (MD: 0.14, 95% CI [0.10~0.18], P<0.00001) (**Figure 3D**). Moreover, these complement components in severe NAFLD were significantly higher than those in moderate NAFLD. Serum C3 levels in severe NAFLD were higher than those in moderate NAFLD (MD: 0.45, 95% CI [0.13~0.76], P=0.005) (**Figure 4A**); serum ASP levels in severe NAFLD were higher than those in moderate NAFLD (MD: 7.83, 95% CI [6.32~9.34], P<0.00001) (**Figure 4B**); serum C5 levels in severe NAFLD were higher than those in moderate NAFLD (MD: 8.43, 95% CI [5.77~11.09], P<0.00001) (**Figure 4C**); serum CFB levels in severe NAFLD were higher than those in moderate NAFLD (MD: 0.11, 95% CI [0.07~0.15], P<0.00001) (**Figure 4D**). Since the included studies did not provide raw data that could be compared according to the severity of the disease, we could not obtain the relationship between C4, CFD and the severity of NAFLD.

### Subgroup analysis and regression analysis

Due to the obvious heterogeneity observed in assessing the relationship between C3 levels and the risk of NAFLD, we conducted subgroup analysis according to region and type of study. The summary results showed that high heterogeneity was observed in Eastern (I^2 =^ 100%, P=0.0007) and Western countries (I^2 =^ 83%, P=0.005) (**Supplementary Figure 1A**). High heterogeneity was identified again in the research design types (**Supplementary Figure 1B**). Meta-regression showed that different research types may be the source of the heterogeneity in the meta-analysis (**Supplementary Table 5**).

### Publication bias

Among the fourteen studies included to assess the relationship between C3 and NAFLD, the asymmetric funnel plot indicated that there might be publication bias (**Supplementary Figure 2**). Egger’s test indicated that there was no statistically significant publication bias (P=0.115) (**Supplementary Figure 3A**). We further performed trimming and filling methods for sensitivity analysis, and the results confirmed that the conclusion was not reversed (MD=2.073, 95% CI [1.532, 2.615], P=0.000) (**Supplementary Figure 3B**).

## Discussion

**Figure 5:**
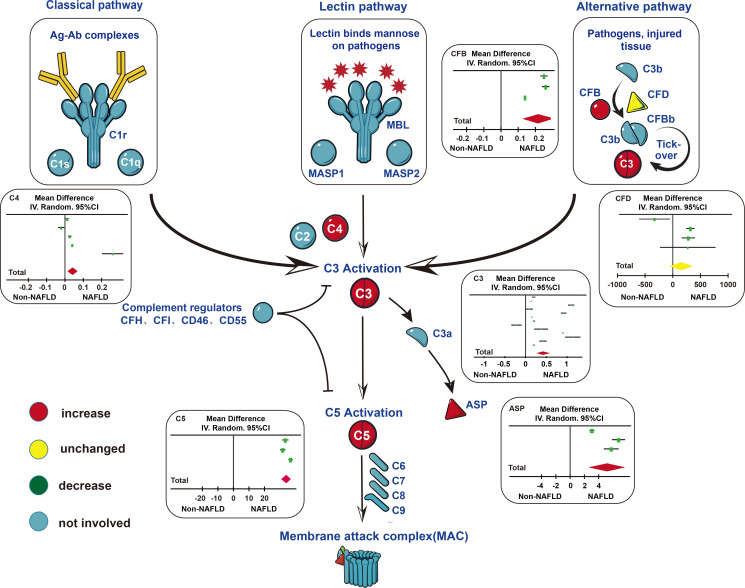
Changes of complement components in three main pathways of complement system activation. In the classical pathway and lectin pathway, serum C4 levels in patients with NAFLD were higher than those in the control group. In the alternative pathway, serum CFB levels in patients with NAFLD were higher than those in the control group. In the subsequent common pathway, serum C3, C5, ASP levels in patients with NAFLD were higher than those in the control group. Abbreviations: ASP, acylation stimulating protein; C1r, complement component 1 r; C1s, complement component 1 s; C1q, complement component 1 q; C2, complement component 2; C3, complement component 3; C4, complement component 4; C5, complement component 5; C6, complement component 6; C7, complement component 7; C8, complement component 8; C9, complement component 9; CFB, complement component B; CFD, complement component D; CFH, complement component H; CFI, complement component I; CD46, Membrane cofactor protein (MCP); CD55, Decay-accelerating factor (DAF); MAC, membrane attack complex; MASP, mannose-binding lectin-associated serine protease; MBL, mannose-binding lectin.

In the 18 studies included, we can conclude that the levels of complement components (C3, ASP, C4, C5, and CFB) in patients with NAFLD were higher than those in healthy controls. Compared to patients with mild NAFLD, the levels of complement components (C3, ASP, C5, and CFB) in patients with moderate NAFLD were significantly increased. Similarly, the levels of complement components (C3, ASP, C5, and CFB) in patients with severe NAFLD were higher than patients with moderate NAFLD (**Figure 5**).

Complement components mainly produced by the liver play an important role in regulating inflammation and resisting pathogens in the human body. Complement activation and inactivation are closely related to many autoimmune diseases (12, 15). As the core component of complement activation, immune defense and immune regulation, C3a generated by C3 can induce eosinophils and mast cells, indirectly activate neutrophils, produce multiple immune responses and eliminate pathogens (52). The three major activation pathways of complement include the traditional pathway of immune complex activation, the mannan-binding lectin pathway and the bypass pathway, which are involved in the occurrence and progression of NAFLD to varying degrees. While the specific mechanism is not clear, the severity of NAFLD is associated with the accumulation of C3 active products around fatty degenerated hepatocytes (30). Complement activation marker C3a is associated with hepatic steatosis and hepatocyte injury in individuals with heavy alcohol and severe obesity (20). In addition, the plasma level of C3a is higher in NAFLD subjects than in healthy controls, and C3b deposition is more abundant (13, 19). The levels of C3c and properdin increase with the degree of hepatic lobule inflammation in NASH subjects (30). ASP is a derivative lipid hormone produced by the interaction of CFB and CFD. As the center of lipid and glucose metabolism regulation, triglycerides (TGs), the main component of liver fat, are mainly derived from the esterification of free fatty acids (FFAs). ASP dysfunction or dysfunction of transport pathways will reduce the uptake of FFAs by adipocytes, increase the flow of FFAs to the liver, and eventually lead to hypertriglyceridemia, lipid metabolism disorder and liver steatosis (19). Studies have shown that the contents of C4 and alanine aminotransferase are highly correlated with the histological activity index, although no relationship between serum C4 levels and fibrosis has been found (53). In a mouse model of alcoholic liver disease, C5 was involved in the regulation of serum TGs and cholesterol and promote liver steatosis and inflammation by mediating the expression of interleukin (IL)-1β (7, 54). CFB is one of main proteases in the complement alternative pathway. After inhibiting the activity of CFB, the activation of complement alternative pathway in the liver will be significantly weakened (55, 56).

In a liver biopsy study, authors confirmed that the extensive deposition of C3 and C4d in liver tissue patients with NAFLD was related to excessive fat accumulation, hepatocyte apoptosis and liver neutrophil isolation (13). After adjusting for mixed factors such as age and sex, the results showed that approximately 15% of the relationship between insulin resistance and plasma C3 could be explained by liver fat accumulation (represented by plasma alanine transaminase [ALT] levels) (18). Similarly, after adjusting for potential confounders in a cross-sectional study, serum C3 levels were independently associated with NAFLD and AFLD, and increased C3 levels were associated with disease severity, which was consistent with our findings (15). However, another study found that the circulating CFD level of NAFLD subjects decreased, and the possibility of NAFLD in subjects with low CFD level was much higher than that in subjects with high CFD level in regression analysis (32).

The number of research samples we chose varied from 76 to 7540, and the gap in quantity may be one of the sources of heterogeneity. Regression analysis showed that different research types may inevitably lead to heterogeneity. Among the included studies, seven studies were based on the guidelines formulated by the Chinese Medical Association, six were based on abdominal ultrasound, and only one was based on pathological examination. Different diagnostic criteria may also cause heterogeneity. The studies we included involved China, Italy, and Turkey. Samples from different regions may also be one of the sources of heterogeneity. In addition, in the included studies, the earliest research can be traced back to 2003, the latest research can be traced back to 2018, and the age span of the subjects is more than 30 years (from 33 to 65 years old), which may lead to heterogeneity. Sensitivity analysis showed that no single study can reverse the combined MD or standard mean difference (SMD), indicating that the results of our analysis are reliable.

Our study has some limitations. First, the diagnostic criteria for NAFLD included in the study are not uniform, which may inevitably lead to heterogeneity. Second, the number of cases in some studies was too small (no more than 100 cases), and only 3 to 4 studies assessed the association of complement components with disease severity, however, no significant publication bias was found, and the sensitivity analysis remained solid. Moreover, C4 and CFD cannot be compared according to the severity of the disease due to insufficient original data. Third, most of the studies were conducted on the Chinese population, and ethnic and regional coverage was not wide enough. We performed a subgroup analysis on this and found that ethnic and region coverage were not sources of heterogeneity. Fourth, the included studies were basically cross-sectional studies or case−control studies. Due to the lack of prospective studies, we cannot determine the causal relationship between these complement components and the occurrence and development of NAFLD. More and higher-quality prospective studies in more ethnic groups are needed to better explain the association of complement components with NAFLD.

## Conclusion

Our study shows that complement components (C3, ASP, C4, C5, and CFB) are almost all related to the risk and severity of NAFLD, indicating that the above complement factors are expected to be potential biomarkers for clinical diagnosis and treatment of NAFLD, which is worthy of further study in the future.

## Data availability statement

The original contributions presented in the study are included in the article/**Supplementary Material**. Further inquiries can be directed to the corresponding authors.

## Author contributions

XF, PL, LG and JJZ designed the study and assessed the quality of the study. JBZ performed the analysis and wrote the manuscript. XF, XW, PL, YW, YC, JH, YS, YL and ZW revised the manuscript. All authors contributed to the article and approved the submitted version.
