# Supplementary material for: Association of complement components with the risk and severity of NAFLD: A systematic review and meta-analysis
Source: Front Immunol. 2022 Dec 7;13:1054159. doi: 10.3389/fimmu.2022.1054159 (PMC9782972; doi:10.3389/fimmu.2022.1054159)
Supplement: Supplementary file 1 [file DataSheet_1.docx]

**Supplementary Figures**


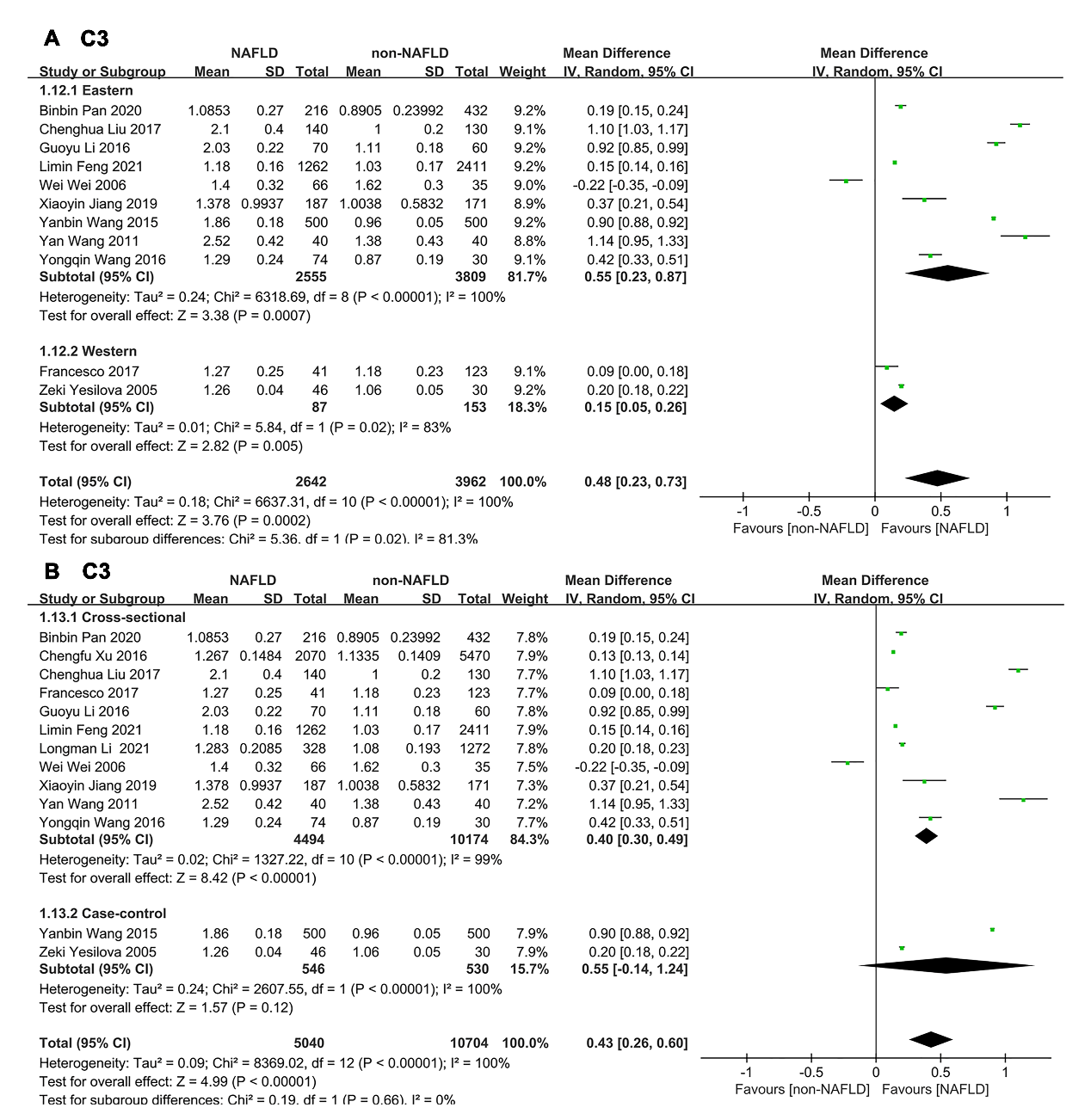


**Supplementary Figure 1. Subgroup analysis on the deviation of C3 between NAFLD and non-NAFLD.**

**(A)** Subgroup analysis of the difference between NAFLD and non-NAFLD patients based on location. **(B)** Subgroup analysis on the difference between NAFLD and non-NAFLD patients based on research type. The forest plots showed the MD value and 95 % CI of NAFLD group compared with non-NAFLD group. Due to the large heterogeneity, a random effect model was used to analyze. Abbreviations: C3, complement component 3; Chi, chi-squared; CI, confidence interval; df, degrees of freedom; IV, Inverse Variance; NAFLD, nonalcoholic fatty liver disease; SD, standard deviation; Tau, tau-squared.


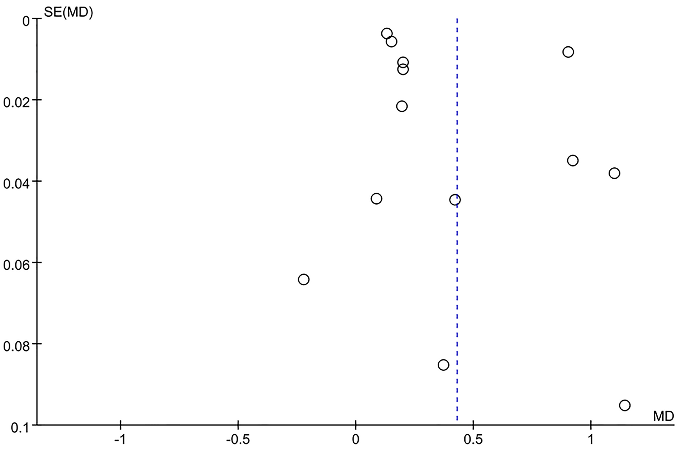


**Supplementary Figure 2. Funnel plot of complement C3.**

This funnel plot is used to evaluate publication bias. Each circle represents a study, the transverse axis represents the effect size MD, and the longitudinal axis represents the standard error. Abbreviations: C3, complement component 3; MD, mean difference; SE, standard error.


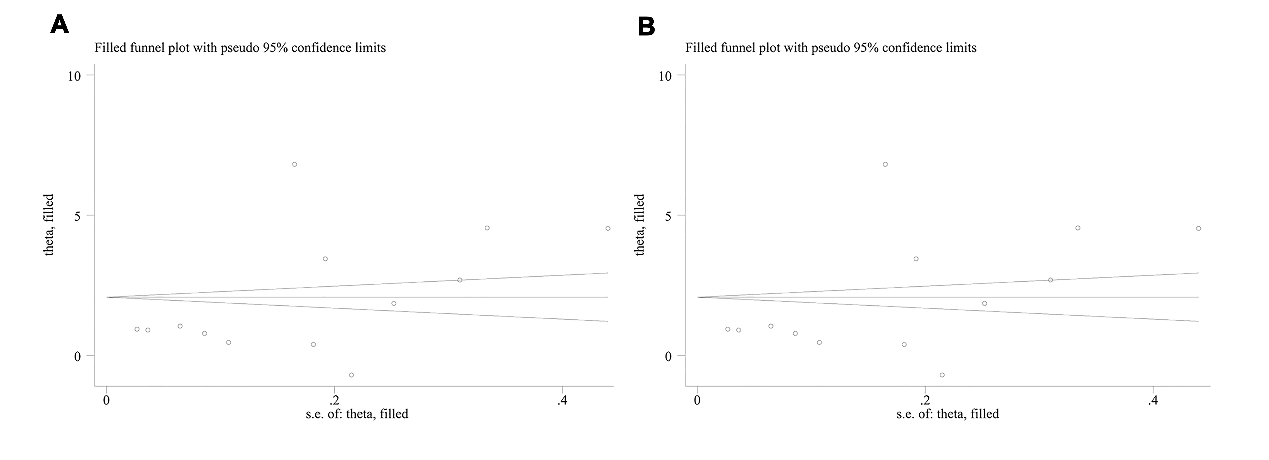


**Supplementary Figure 3. Funnel plots.**

**(A)** Egger’s test of C3 between NAFLD and non-NAFLD. **(B)** The trim-and-fill computation was used to test the publication bias on C3.

**Supplementary Tables**

**Supplementary Table 1** | Cross-Sectional/Prevalence Study Quality

|  |  | Define the source of information (survey, record review) | List inclusion and exclusion criteria for exposed and unexposed subjects (cases and controls) or refer to previous publications | Indicate time period used for identifying patients | Indicate whether or not subjects were consecutive if not population-based | Indicate if evaluators of subjective components of study were masked to other aspects of the status of the participants | Describe any assessments undertaken for quality assurance purposes (e.g., test/retest of primary outcome measurements) | Explain any patient exclusions from analysis | Describe how confounding was assessed and/or controlled. | If applicable, explain how missing data were handled in the analysis | Summarize patient response rates and completeness of data collection | Clarify what follow-up, if any, was expected and the percentage of patients for which incomplete data or follow-up was obtained |  |
| --- | --- | --- | --- | --- | --- | --- | --- | --- | --- | --- | --- | --- | --- |
| Binbin Pan | 2020 | Yes | Yes | Yes | Yes | Unclear | Yes | Yes | Unclear | No | No | Unclear | 6 |
| Chengfu Xu | 2016 | Yes | Yes | Yes | Yes | Unclear | Yes | Yes | Yes | Unclear | No | Unclear | 7 |
| Chenghua Liu | 2017 | Yes | Yes | Yes | No | Unclear | Yes | Unclear | No | Unclear | Yes | Unclear | 5 |
| Francesco | 2017 | Yes | Yes | Yes | Yes | Unclear | Yes | Yes | Yes | Unclear | No | Unclear | 7 |
| Guoyu Li | 2016 | Yes | Yes | Yes | No | Unclear | Yes | Unclear | No | Unclear | Yes | Unclear | 5 |
| Limin Feng | 2021 | Yes | Yes | Yes | Yes | Unclear | Yes | Yes | Yes | Unclear | Yes | Unclear | 8 |
| Longman Li | 2021 | Yes | Yes | Yes | Yes | Unclear | Yes | Yes | Yes | Unclear | Yes | Yes | 9 |
| Wei Wei | 2006 | Yes | Yes | Yes | Unclear | Unclear | Yes | No | No | No | Yes | Unclear | 5 |
| Xiaoyin Jiang | 2019 | Yes | Yes | Yes | Yes | Unclear | Yes | Yes | Unclear | Unclear | Yes | Unclear | 7 |
| Yan Wang | 2011 | Yes | Yes | Yes | Yes | Unclear | Yes | Unclear | Unclear | Unclear | Unclear | Unclear | 5 |
| Yanbin Wang | 2015 | Yes | Yes | Yes | Yes | Unclear | Yes | Unclear | No | No | No | No | 5 |
| Yongan Liu | 2013 | Yes | Yes | Yes | No | Unclear | Yes | Unclear | No | Unclear | Yes | Unclear | 5 |
| Yongqin Wang | 2016 | Yes | Yes | Yes | Yes | Unclear | Yes | Unclear | Yes | Unclear | Yes | Unclear | 7 |
| Zeki Yesilova | 2005 | Yes | Yes | Yes | Yes | Unclear | Yes | Yes | Yes | Unclear | Yes | Unclear | 8 |
| Jinhua Zhang | 2021 | Yes | Yes | Yes | Yes | Unclear | Yes | Yes | Yes | Unclear | Unclear | Unclear | 7 |
| Yingying Gu | 2022 | Yes | Yes | Yes | Yes | Unclear | Yes | Yes | Yes | Unclear | Yes | Yes | 9 |
| Yun Qiu | 2019 | Yes | Yes | Yes | Yes | Unclear | Yes | Yes | Yes | Unclear | Unclear | Unclear | 7 |
| Yusuf Yilmaz | 2011 | Yes | Yes | Yes | Yes | Unclear | Yes | Yes | Yes | Unclear | Unclear | Unclear | 7 |

Article quality was assessed as follows: low quality = 0–3; moderate quality = 4–7; high quality = 8–11.

**Supplementary Table 2** | Complement components were compared between non-NAFLD and NAFLD groups.

|  | Studies | Meta-analysis results | | |  | Heterogeneity test | | |
| --- | --- | --- | --- | --- | --- | --- | --- | --- |
|  |  | MD | 95% CI | P value |  | Effect models | I^2^ | P^a^ value |
| C3 | 13 | 0.43 | (0.26, 0.60) | <0.00001 |  | Random | 100% | <0.00001 |
| ASP | 3 | 5.17 | (2.57, 7.77) | <0.0001 |  | Random | 97% | <0.00001 |
| C4 | 5 | 0.04 | (0.02, 0.07) | 0.002 |  | Random | 97% | <0.00001 |
| C5 | 3 | 34.03 | (30.80, 37.27) | <0.00001 |  | Random | 91% | <0.0001 |
| CFB | 3 | 0.22 | (0.13, 0.31) | <0.00001 |  | Random | 99% | <0.00001 |

Abbreviation: ASP, acylation stimulating protein; C3, complement component 3; C4, complement component 4; C5, complement component 5; CFB, complement factor B; CI, confidence interval; MD, mean difference; NAFLD, nonalcoholic fatty liver disease.

**Supplementary Table 3** | Complement components were compared between mild and moderate NAFLD.

|  | Studies | Meta-analysis results | | |  | Heterogeneity test | | |
| --- | --- | --- | --- | --- | --- | --- | --- | --- |
|  |  | MD | 95% CI | P value |  | Effect models | I^2^ | P value |
| C3 | 4 | 0.27 | (0.21, 0.34) | <0.00001 |  | Random | 0% | 0.91 |
| ASP | 3 | 8.28 | (4.86, 11.70) | <0.00001 |  | Random | 87% | 0.0006 |
| C5 | 3 | 9.83 | (6.22, 13.43) | <0.00001 |  | Random | 59% | 0.09 |
| CFB | 2 | 0.14 | (0.10, 0.18) | <0.00001 |  | Random | 43% | 0.18 |

Abbreviation: ASP, acylation stimulating protein; C3, complement component 3; C5, complement component 5; CFB, complement factor B; CI, confidence interval; MD, mean difference; NAFLD, nonalcoholic fatty liver disease.

**Supplementary Table 4** | Complement components were compared between moderate and severe NAFLD.

|  | Studies | Meta-analysis results | | |  | Heterogeneity test | | |
| --- | --- | --- | --- | --- | --- | --- | --- | --- |
|  |  | MD | 95% CI | P value |  | Effect models | I^2^ | P value |
| C3 | 4 | 0.45 | (0.13, 0.76) | 0.005 |  | Random | 92% | <0.00001 |
| ASP | 3 | 7.83 | (6.32, 9.34) | <0.00001 |  | Random | 14% | 0.31 |
| C5 | 3 | 8.43 | (5.77, 11.09) | <0.00001 |  | Random | 0% | 0.57 |
| CFB | 2 | 0.11 | (0.07, 0.15) | <0.00001 |  | Random | 0% | 0.46 |

Abbreviation: ASP, acylation stimulating protein; C3, complement component 3; C5, complement component 5; CFB, complement factor B; CI, confidence interval; MD, mean difference; NAFLD, nonalcoholic fatty liver disease.

**Supplementary Table 5** | Meta-regression analysis of potential sources of heterogeneity.

| Covariates | Coefficients | Lower bound | Upper bound | Std. error | P value |
| --- | --- | --- | --- | --- | --- |
| Sample size | -0.000205 | -0.000853 | 0.0004434 | 0.0002946 | 0.501 |
| Study design | 4.243975 | 1.641779 | 6.846171 | 1.182287 | 0.004 |
| Year | -0.0868501 | -0.358343 | 0.1846428 | 0.1233506 | 0.496 |
